# A novel strategy for identifying biomarker in serum of patient with COVID-19 using immune complex

**DOI:** 10.1038/s41392-022-00909-z

**Published:** 2022-02-28

**Authors:** Fugang Duan, Yifan Wang, Taoyu Chen, Zhu Zhu, Meng Yu, Hui Dai, Shangen Zheng, Yinying Lu, Tingting Li, Xiaoyan Qiu

**Affiliations:** 1 Department of Immunology, School of Basic Medical Sciences, Peking University Health Science Center, Beijing, 100191 China; 2 NHC Key Laboratory of Medical Immunology, Peking University Health Science Center, Beijing, 100191 China; 3 Key Laboratory of Molecular Immunology, Chinese Academy of Medical Sciences, Beijing, 100191 China; 4 Department of Biomedical Informatics, School of Basic Medical Sciences, Peking University Health Science Center, Beijing, 100191 China; 5 Department of Transfusion, General Hospital of Central Theater Command of the People’s Liberation Army, Wuhan, Hubei 430070 China; 6 Comprehensive Liver Cancer Center, The 5th Medicine Center of PLA General Hospital, Beijing, 100039 China

**Keywords:** Infectious diseases, Predictive markers

**Dear Editor**,

COVID-19 (Coronavirus Disease 2019) is a disease caused by the single-stranded sense RNA virus SARS-CoV-2 (severe acute respiratory syndrome coronavirus 2), which has caused a global public health crisis.^1^ However, so far no effective serum marker has been found as a biomarker for COVID-19 diagnosis, except that viral nucleic acid detection is an effective evidence of SARS-CoV-2 infection.

Usually, whether it is a systemic disease or a disease occurring in an organ, it will be accompanied by changes in serum protein profile. Changes in human plasma proteins can be used as biological indicators of pathological changes caused by many diseases.^2^ In particular, the current high-throughput proteome technology can quickly find candidate markers of disease.^3^ Recently, several candidate biomarkers in plasma of patients of COVID-19, such as S100A8/A9, CFI et al, were found by the proteomics technology.^4^ However, most studies that try to find the characteristic biomarkers investigate whole serum proteins from the patients of COVID-19. The protein in the whole serum may not fully reflect the characteristics of COVID-19 disease. It is known that the immune system can sense the infection of any pathogen and abnormal changes of the body, and then produce specific antibodies to bind and remove pathogens or some endogenous antigens caused by tissue damage (such as denatured proteins or some intracellular protein). Theoretically, circulating antibodies may carry disease-related antigen information in the form of immune complexes, moreover, different classes of antibodies may indicate different antigen infections. Therefore, we make assumptions that using immune complexes to find COVID-19 markers may be a more accurate strategy.

Here, to test our hypothesis, we respectively collected 9 sera samples from COVID-19 patients 2 weeks after the recovery period and 9 sera samples from healthy donors as control, then the immune complexes are purified by different affinity columns, including IgG complexes purified by protein G columns, IgA complexes purified by Jacalin columns, and IgM complexes purified by anti-human IgM columns (Fig. 1a). Next, the purified immune complexes are analyzed by mass spectrometry to obtain antibody-bound disease-related antigen profile. Mass spectrometry allows a thorough mapping of compositions within samples, whose data can be compared to reflect the differences of proteins between groups.^5^ After normalizing the mass spectrometry data and comparing to the healthy controls, we found that in serum of COVID-19 patients 2 weeks after recovery period, 26 proteins bound to IgA were increased and 1 protein was decreased (Fig. 1b), 10 proteins bound to IgG were increased and 1 protein were decreased (Fig. 1c) and 2 proteins bound to IgM increased (Fig. 1d). Moreover, as expected, either IgM complexes, IgG complexes and IgA complexes displayed their own unique antigen profile, respectively, compared with the healthy controls. We also performed GO and KEGG enrichment to understand where their biological properties lie in Supplementary Fig 1. Among them, the functions of IgA-bound proteins include immunoglobulin mediated immune response, the complement system, and the coagulation cascade. The functions of IgG-bound proteins include complement activation, immunoglobulin mediated immune response, and so on, while functions of proteins bound by IgM enriching in complement activation and so on. Next, we are more interested in those proteins that only bind to IgM, IgA and IgG during the recovery period (2 weeks) of COVID-19, but not in the healthy group. We really find some proteins that exist in patients with COVID-19 and not in healthy individuals, such as Carbonic anhydrase I (CA1) showed in IgA-complex and Leucine-rich-alpha-2-glycoprotein 1(LRG1) in IgG-complex (Supplementary Fig. S2a, b). Among all the samples, CA1 and LRG1 each have 4 and 13 peptides identified by mass spectrometry indicated by 7 and 75 peptide-spectrum matches respectively.

**Fig. 1:**
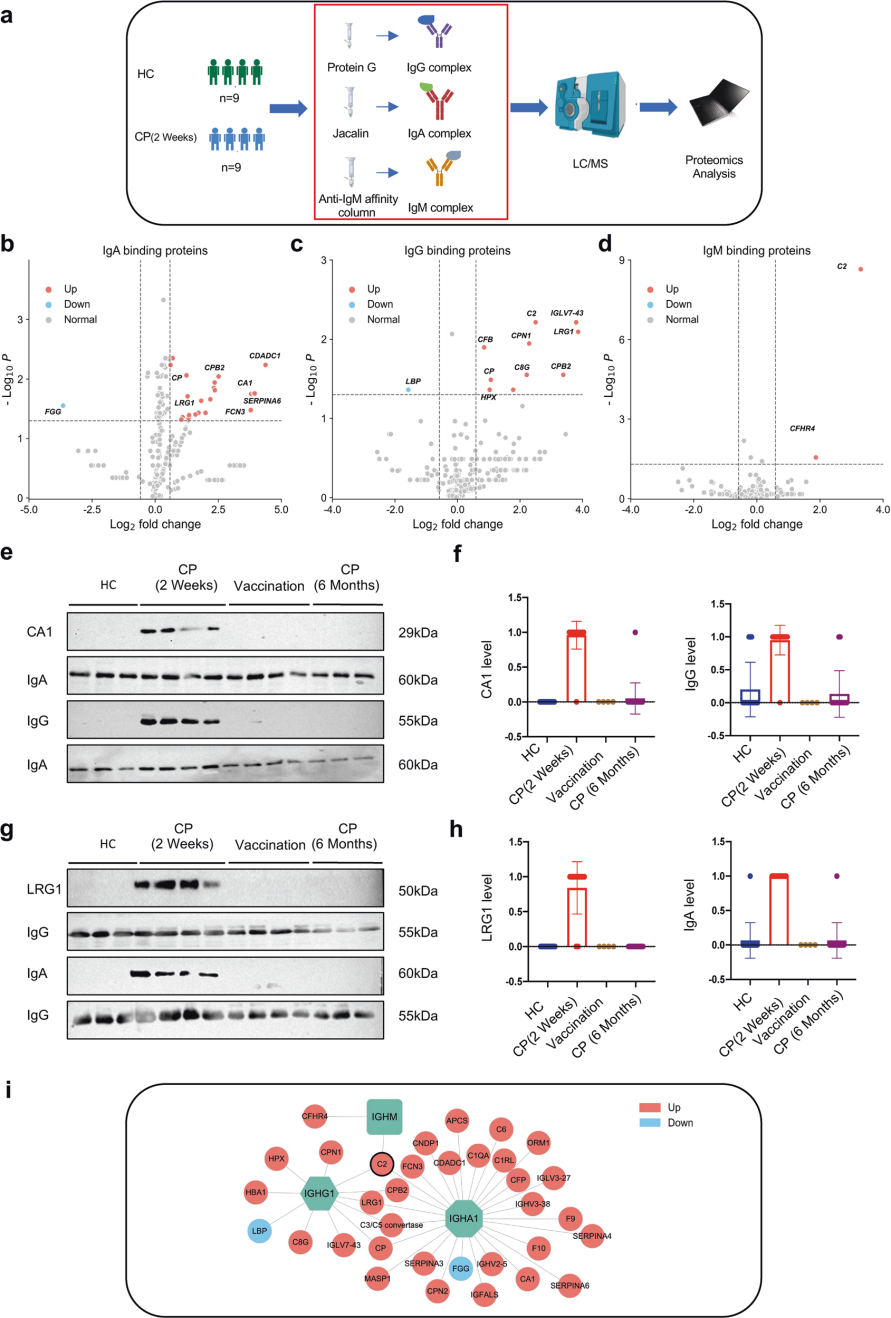
The immunoglobulin complex is a biomarker in COVID-19. **a** The workflow for processing proteomic data, including plasma separation, affinity chromatography, LC-MS/MS, and data analysis (Picture produced by Biorender). The volcano map shows that IgA complex (**b**), IgG complex (**c**), and IgM complex (**d**) have significantly different proteins. **e**, **f** Western blot detection of IgA-bound CA1 and Jacalin-bound IgG in different groups (healthy control group, COVID-19 recovery period (2 weeks) group, vaccination group, COVID-19 recovery period (6 months) group). **g**, **h** Western blot illustrates the binding of IgG and LRG1 and the binding of Protein G and IgA in the healthy group, the COVID-19 recovery period (2 weeks) group, the COVID-19 vaccination group and the COVID-19 recovery period (6 months) group. **i** The network diagram shows the potential biomarkers of immunoglobulin complex in COVID-19

Subsequently, we expanded the serum samples of COVID-19 patients in 2 weeks after the recovery period to 20 cases, and further collected 20 serum samples of COVID-19 patients in the recovery period of 6 months and 4 serum samples from healthy volunteers inoculated with SARS-CoV-2 inactivated vaccine, to confirm whether these proteins that exist only in patients with COVID-19 can be used as specific markers for patients with COVID-19 (Supplementary Table 1). We choose both IgA-bound CA1 and IgG-bound LRG1 as models, and found that CA1 bound to IgA displayed in COVID-19 patients recovered within 2 weeks, but not in any healthy, vaccinated healthy subjects or COVID-19 patients 6 months after recovery (Fig. 1e, f). Similarly, LRG1 bound to IgG only displayed in COVID-19 patients recovered within 2 weeks, but not in any healthy, vaccinated subjects or COVID-19 patients 6 months after recovery (Fig. 1g, h). Interestingly, CA1 and LRG1 did not increase in whole serum, suggesting the role of the complex in the immune response (Supplementary Fig. 3). CA1 is a cytoplasmic isomer of a member of the α-CA family of mammals, responsible for maintaining the steady-state of pH during the physiological and pathological processes of the organism. LRG1 is a new angiogenesis regulator, which mediates its effects by regulating TGF-β signaling. Moreover, recent studies have found that LRG1 can be used as a biomarker for the recovery period of COVID-19, which also supports our findings.

In addition, we unexpectedly found that IgG peptide showed in Jacalin-purified IgA-complex, moreover, a 55 kDa band (like the IgG) usually showed under the Jacalin- purified IgA, in the COVID-19 patients 2 weeks after recovery period, the finding suggested that there may be an IgA-IgG complex in these COVID-19 patients. In order to verify our hypothesis, we first used anti-IgA and anti-IgG antibodies to detect Jacalin-purified IgA. As shown in Supplementary Fig. 4a, Jacalin-purified IgA contains IgG. Moreover, we have identified the subclass of the IgG included all of IgG1, IgG2, IgG3, and IgG4 (Supplementary Fig. 4b). Similarly, the IgG was found to be shown in all of 20 COVID-19 patients 2 weeks after the recovery period (Fig. 1e, f). Next, we further isolated the IgG from Jacalin-bound IgA-complexes by protein G from the serum of patients with COVID-19 2 weeks after the recovery period and identified the protein profile of the IgG-bound protein by mass spectrometry (Supplementary Fig. 4c). As shown in Supplementary Fig. 4d, e, the IgG-bound protein included 5 different proteins compared to Protein G-bound IgG. Next, we also tested if there is IgA in Protein G-purified IgG-complexes from COVID-19 patients, as expected, the IgG-complexes also included IgA that can be found in the COVID-19 patients 2 weeks after the recovery period (Fig. 1g, h).

In this study, we hypothesized according to the principles of immunology that the circulating immune complexes can carry specific disease-related proteins, which can be identified by affinity purification combined with protein mass spectrometry (also named as Immune Complex Omics). In summary, our research shows that the immune complexes can be used to discover highly specific COVID-19 biomarkers (Fig. 1i). Moreover, the strategy can be extended to searches for markers related to other diseases. Our research results show that the proteins bound by these immune complexes are mainly intracellular proteins. It is suggested that the immune complex binding protein in these disease states may indicate the degree of tissue or cell damage, so as to gain insight into its mechanism.

## Supplementary information

R2- letter Supplementary information clean

Supplementary Table 1

## Data Availability

All data that support the findings of this study are available from the corresponding author upon reasonable request.
