# Supplementary material for: A novel strategy for identifying biomarker in serum of patient with COVID-19 using immune complex
Source: Signal Transduct Target Ther. 2022 Feb 28;7:63. doi: 10.1038/s41392-022-00909-z (PMC8882717; doi:10.1038/s41392-022-00909-z)
Supplement: Supplementary file 1 — R2- letter Supplementary information clean [file 41392_2022_909_MOESM1_ESM.docx]

Supplementary Materials for

A novel strategy for identifying biomarker in serum of patient with COVID-19 using immune complex

Fugang Duan^1,2,3^*, Yifan Wang^1,2,3^*, Taoyu Chen^4*^, Zhu Zhu^1,2,3^*, Meng Yu^1,2,3^, Hui Dai^1,2,3^, Shangen Zheng^5^, Yinying Lu^6#^, Tingting Li^4#^, Xiaoyan Qiu^1,2,3^^#^

1 Department of Immunology, School of Basic Medical Sciences, Peking University Health Science Center, Beijing, 100191, China;

2 NHC Key Laboratory of Medical Immunology, Peking University Health Science Center, Beijing, 100191 China;

3 Key Laboratory of Molecular Immunology, Chinese Academy of Medical Sciences, Beijing, 100191, China;

4 Department of Biomedical Informatics, School of Basic Medical Sciences, Peking University Health Science Center, Beijing 100191, China;

5 Department of Transfusion, General Hospital of Central Theater Command of the People's Liberation Army, Wuhan, Hubei, 430070,China;

6 Comprehensive Liver Cancer Center, The 5th Medicine Center of PLA General Hospital, Beijing, 100039, China;

* These authors contributed equally

# Correspondence: Xiaoyan Qiu ([qiuxy@bjmu.edu.cn](mailto:qiuxy@bjmu.edu.cn)), Tingting Li (litt@hsc.pku.edu.cn ), Yinying Lu( luyinying1973@163.com ).

This PDF file includes:

Materials and Methods

Supplementary Fig 1 to 4

**Materials and Methods**

**Serum sample preparation**

The blood for COVID-19 recovery period of 2 weeks and the COVID-19 recovery period of 6 months came from The 5th Medicine Center of PLA General Hospital. All COVID-19 patients tested positive for respiratory tract RT-PCR as infection. Blood samples are collected in standard vacuum tubes using standard venipuncture protocols. Centrifuge at 3000 rpm for 10 minutes to extract the serum. Next, the serum sample was inactivated at 56°C for 30 min and then stored at -80°C. Disease severity was defined as moderate, severe, and critical, according to the 'Diagnosis and Treatment Protocol of COVID-19 (the 7th Tentative Version)' by the National Health Commission of China issued on 3 March 2020(<http://www.nhc.gov.cn/yzygj/s7653p/202003/46c9294a7dfe4cef80dc7f5912eb1989.shtml>). All relevant experiments were approved by the Research Ethics Committee of The 5th Medicine Center of PLA General Hospital.

**Immune complex purification**

IgA complex purification: Follow the manufacturer's instructions. Firstly, Jacalin (#6561, BioVision) was mixed with serum and incubated at 4°C for 1 hour. Release the flow-through to the column. Then wash the column 4-5 times with 5X volume of PBS containing 0.5 M NaCl and 4-5 times with PBS. Elute immune complexes with elution buffer (0.1 M melibiose or 0.1 M alpha-D-galactose in PBS). Change the buffer to PBS by using a 3 kDa Amicon Ultra centrifugal filter (Merck Millipore). Use Nanodrop to measure the IgA concentration and store the sample at -20°C.

IgG complex purification: The serum was incubated with a Protein G Sepharose column (#17-0618-02, GE Healthcare) at 4°C for 1 h. Then wash the Sepharose column of protein G-IgG with PBS. Finally, 0.1 mol/L glycine (pH = 2.4) was used to elute IgG from protein G and terminated to neutral (pH = 7.0). Change the buffer to PBS by using a 3 kDa Amicon Ultra centrifugal filter (Merck Millipore). Use Nanodrop to measure the IgG concentration and store the sample at -20°C.

IgM complex purification: According to the manual, CNBr-Sepharose 4B and anti-human IgM antibody (#2020-01, SouthernBiotech) were used to prepare covalent coupling column. Plasma was incubated with anti IgM affinity column at 4°C for 1 hour. The affinity column was then washed with PBS. Finally, IgM was eluted from anti-IgM affinity column with 0.1 mol / L glycine (pH = 2.4) and terminated in neutral (pH = 7.0). The buffer was changed to PBS using a 3 kDa Amicon ultracentrifugal filter (Merck millipore). Use Nanodrop to measure the IgM concentration and store the sample at -20°C.

**LC-MS/MS**

IgA, IgG and IgM precipitated protein from 9 patient serum samples and 9 patients without prior COVID-19 infections were separated by SDS-PAGE electrophoresis. By collecting more samples for LC-MS/MS we can avoid random hits in a single MS run, improving the efficacy. We then cut the gels and performed liquid chromatography-mass spectrometry/mass spectrometry (LC-MS/MS) for each precipitated protein samples with LTQ ORBITRAP VELOS PRO (Thermo scientific). Briefly, the gels of each protein sample are excised into lanes of different sizes and digested in-gel. The digested peptides are then sent to the high-pressure chromatography system for fractionation prior to mass spectrometry system. Several computational algorithms allow the spectras acquired from fragmented peptides to match peptides that indicate possible proteins in the original sample. We followed the default settings of MaxQuant to produce a final list of possible proteins in each precipitated sample.

**Data Analysis and Enrichment**

We selected the parameter of iBAQ to indicate the abundance of possible proteins in each sample. To compare relative abundance of proteins between samples, we normalized each sample’s iBAQ to make the precipitator proteins, which are IGHA1 (for IgA-precipitated proteins), IGHG1 (for IgG-precipitated proteins), IGHM (for IgM-precipitated proteins) respectively, equal between different samples of the same group, and calculated their logarithms to represent the proteins’ relative abundance. We then plotted volcano plots indicating fold changes between patients recovered from COVID-19 infections and non-infected controls. Fold changes greater than 1.5 and adjusted p-values (adjusted by Benjamin & Hochberg method) less than 0.05 are considered significant differences between groups. We then enriched all significantly increased/decreased proteins against GO terms and KEGG pathways of all human proteins. All procedures a forementioned are carried out in R.

**Western blotting**

The immunoglobulin complex of affinity chromatography was separated by 12.5% SDS-PAGE and transferred to a nitrocellulose membrane. Next, seal with 5% skimmed milk for 1 hour at room temperature. Rabbit anti-human CA1 (#ab108367, abcam), rabbit anti-human LRG1 (#ab178698, abcam), goat anti-human IgA-HRP (#2050, southernbiotech) and goat anti-human IgG-HRP (#2046, southernbiotech) were used as primary antibodies. After washing the membrane with (1×TBST), add appropriate secondary antibody. Next, incubate with the secondary antibody for 1 hour at room temperature. After washing the membrane, add an appropriate amount of Chemiluminescent Substrate (#34580, Thermo scientific). Imaging system (#A43680, Thermo scientific) for luminescence signal detection. In statistics, 1 means a positive band, and 0 means a negative band.


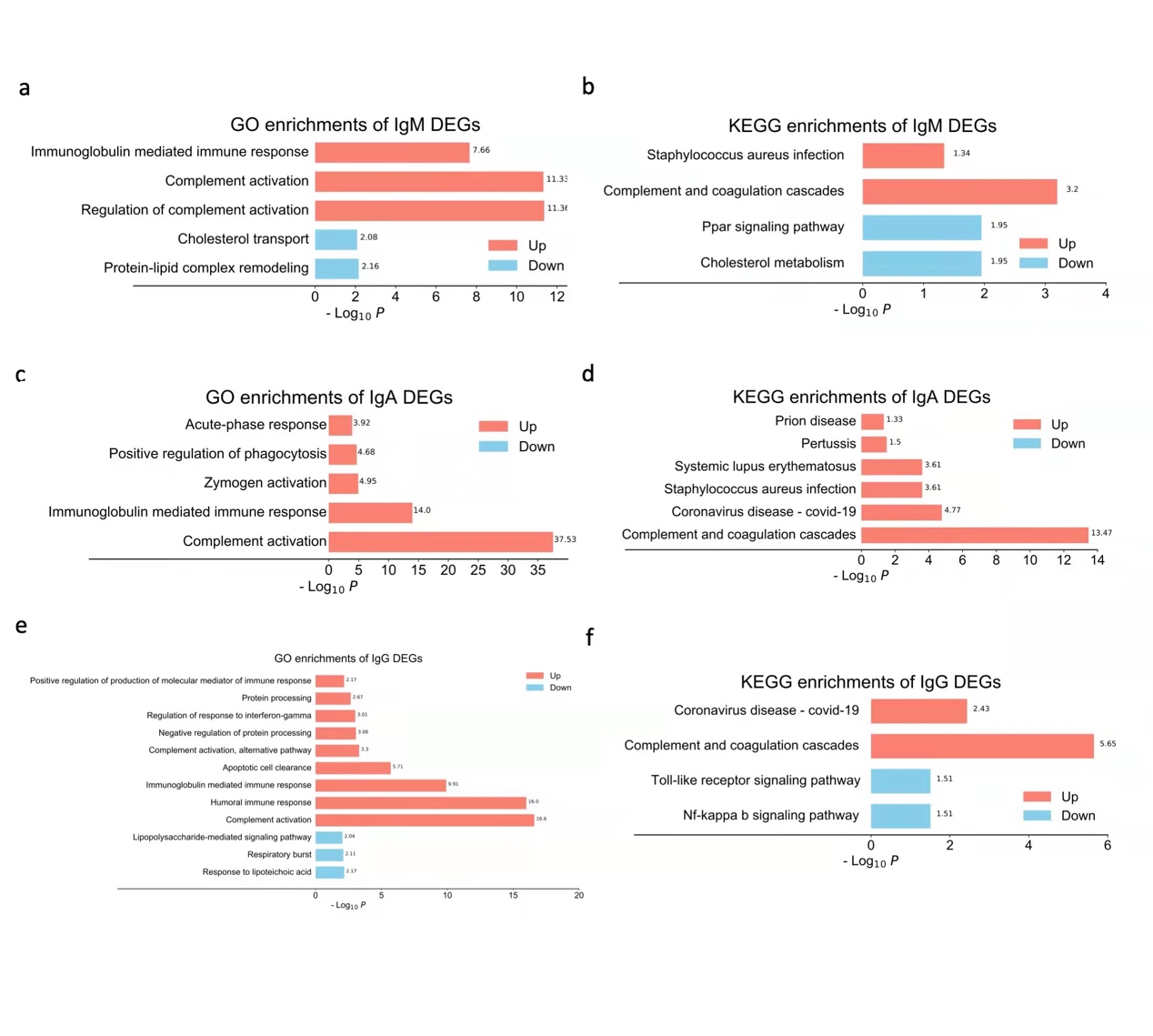


**Supplementary Fig 1.** Enriched all significantly increased/decreased proteins against GO terms and KEGG pathways of all human proteins. (a) and (b) are GO and KEGG enrichment analysis of IgM differentially expressed genes (DEG); (c) and (d) are GO and KEGG enrichment analysis of IgA DEGs; (e) and (f) are GO and KEGG enrichment analysis of IgG DEGs. Red bars indicate increased expression, while blue bars indicate decreased expression.


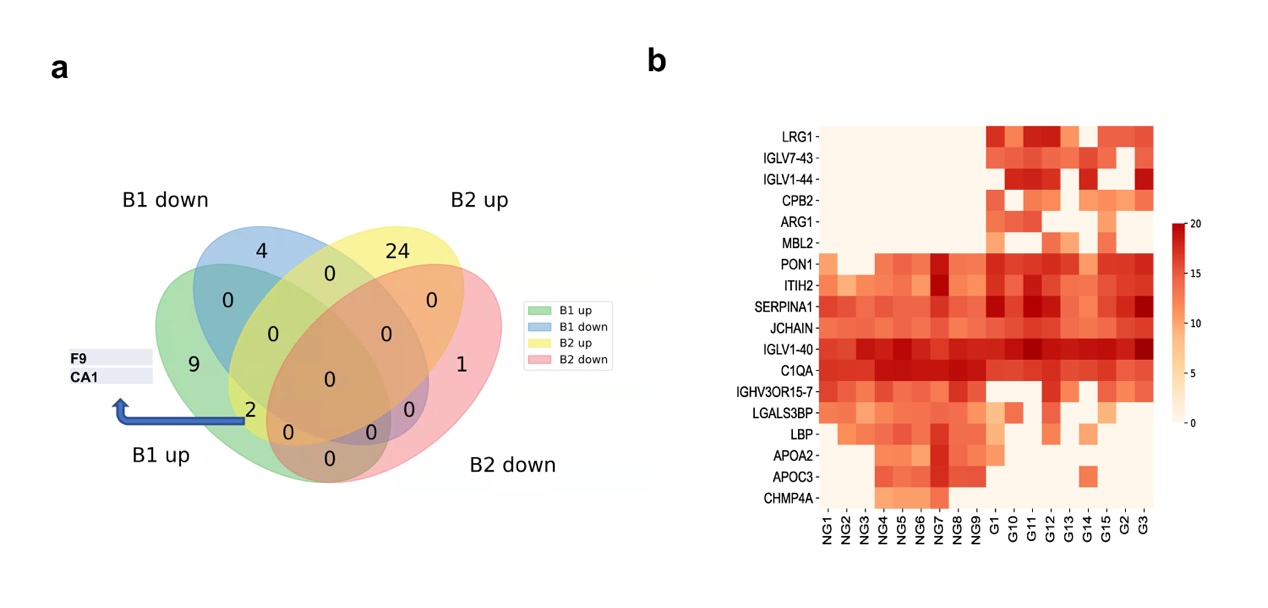


**Supplementary Fig 2.** Validation of the changes in COVID-19 immunoglobulin complex. (a) Venn diagrams of IgA complex protein profiles of two batches of COVID-19 during the 2-week recovery period. (b) Heat map of the protein profile of the IgG complex.


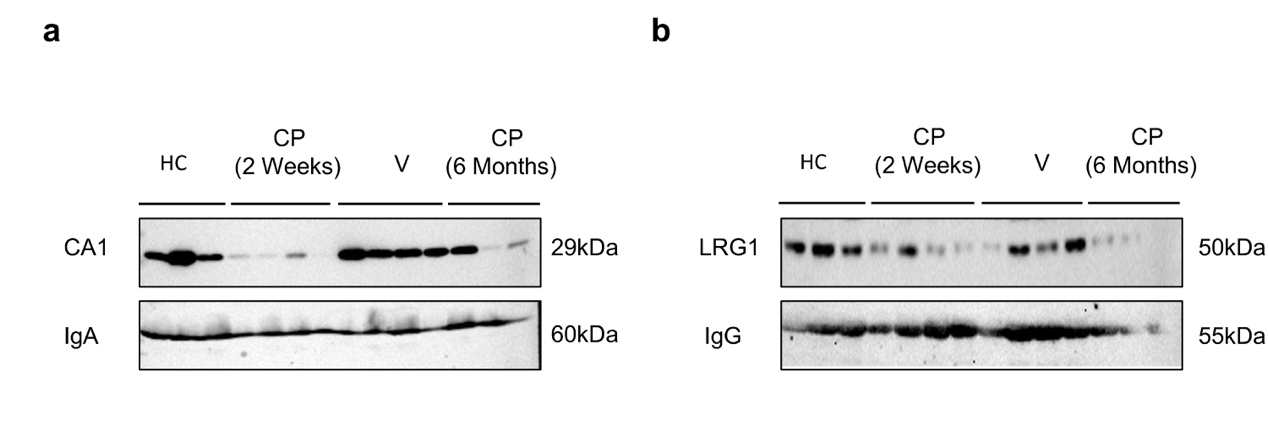


**Supplementary Fig 3.** The expression of CA1, LRG1, IgA and IgG in whole serum. (a)Western blot was used to detect the expression of IgA and CA1 in serum. (b)The expression of IgG and LRG1 in the serum was detected by Western blot. HC: Healthy Control; CP (2 Weeks): COVID-19 Convalescent Phase (2 weeks); V: COVID-19 vaccination; CP (6 Months): COVID-19 Convalescent Phase (6 months).


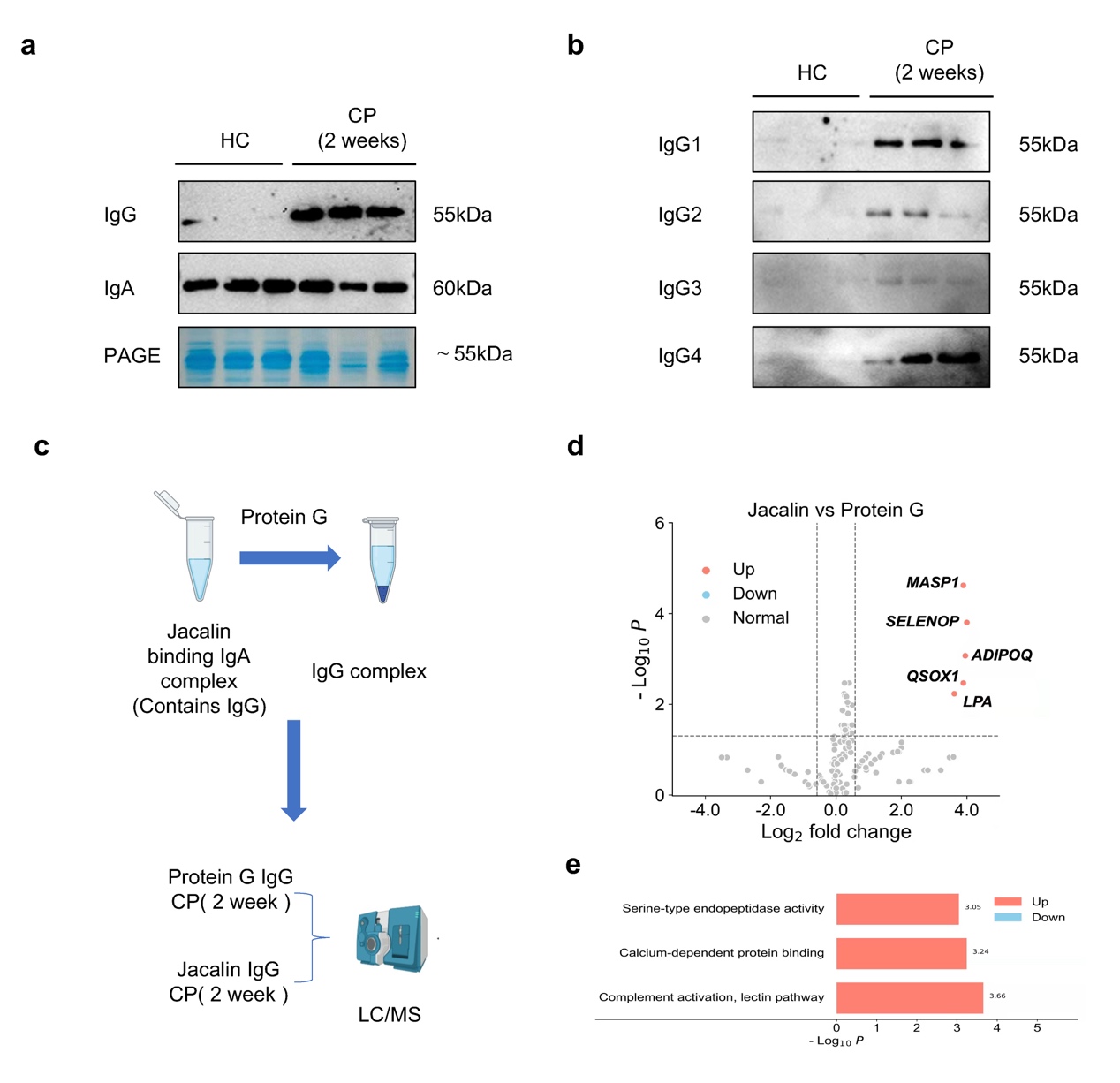


**Supplementary Fig 4.** Jacalin binds to IgG during the COVID-19 recovery period (2 weeks). (a) Jacalin bound IgG was detected during the recovery period of COVID-19 (2 weeks). Jacalin bound IgG is not a non-specific band by SDS-PAGE. (b) The IgG bound by Jacalin contains IgG1, IgG2, IgG3, and IgG4. (c) Schematic diagram of the separation of IgA-IgG complex. (d) The volcano graph shows the difference between IgA-bound IgG and IgG during the 2 weeks of COVID-19 recovery period. (e) Go enrichment analysis of IgA-IgG bound protein and IgG bound protein.
